# Preliminary evidence for an influence of exposure to polycyclic aromatic hydrocarbons on the composition of the gut microbiota and neurodevelopment in three-year-old healthy children

**DOI:** 10.1186/s12887-021-02539-w

**Published:** 2021-02-17

**Authors:** Wei Zhang, Zhongqing Sun, Qian Zhang, Zhitao Sun, Ya Su, Jiahui Song, Bingling Wang, Ruqin Gao

**Affiliations:** 1 Department of Nutrition and Food Hygiene, School of Public Health, Qingdao University, Qingdao, China; 2 Department of Food Hygiene, Qingdao Municipality Center for Disease Control and Prevention, Qingdao Institute of Preventive Medicine, Qingdao, 266033 China; 3 Department of Child Health Care, Huangdao Maternity and Child Health Care Hospital of Qingdao, Qingdao, 266033 China; 4 Department of Environmental Health, Qingdao Municipality Center for Disease Control and Prevention, Qingdao Institute of Preventive Medicine, Qingdao, 266033 China

**Keywords:** Children, Gut microbiota, Polycyclic aromatic hydrocarbons, Neurodevelopment

## Abstract

**Background:**

During the second and third year after birth the gut microbiota (GM) is subjected to important development. The polycyclic aromatic hydrocarbon (PAH) exposure could influence the GM in animal and early postnatal exposure is associated with neurodevelopment disorder in children. This study was designed to explore the possible influence of the polycyclic aromatic hydrocarbons (PAHs) on the composition of the gut microbiota (GM) and neurodevelopment in a sample of 38 healthy children at the age of 3 years.

**Methods:**

A brief development (Gesell Development Inventory, GDI) and behavior test (Child Behavior Checklist, CBCL) were completed on 3-yr-olds and stool samples were collected for 16S rRNA V4-V5 sequencing. The PAH-DNA adduct in the umbilical cord blood and the urinary hydroxyl PAHs (OH-PAHs) at the age of 12 months were measured as pre- and postnatal PAH exposure, respectively.

**Results:**

The most abundant two phyla were *Bacteroidetes* (68.6%) and *Firmicutes* (24.2%). The phyla *Firmicutes*, *Actinobacteria*, *Proteobacteria*, *Tenericutes*, and *Lentisphaerae* were positively correlated with most domain behaviors of the GDI, whereas the *Bacteroidetes*, *Cyanobacteria*, and *Fusobacteria* were negatively correlated. Correspondingly, the phyla *Bacteroidetes*, *Actinobacteria*, and *Fusobacteria* showed positive correlations with most CBCL core and broadband syndromes, whereas the *Firmicutes*, *Verrucomicrobia*, Synergistetes, *Proteobacteria* and *Tenericules* were negatively correlated. The OH-PAH levels were not significantly associated with the *Firmicutes* phylum whereas the *Bacteroidetes*, *Bacteroidia*, and *Bacteroidales* all showed significant negative association with the OH-PAH levels.

**Conclusion:**

The current findings suggest that composition of the GM is associated with neurodevelopment of the child. PAHs seem to change the relative abundance of some taxa (some deleted and some recruited) to counteract the negative effects of the PAHs.

**Supplementary Information:**

The online version contains supplementary material available at 10.1186/s12887-021-02539-w.

## Background

The period of rapid brain growth spans from the 3rd trimester of pregnancy to at least 2 years after birth [1]. This rapid-growth period is a window of opportunity in which foundational processes such as proliferation and migration of glia, myelination of axons, and synaptogenesis are occurring and extremely plastic and can be easily subjected to remodeling in response to some environmental inputs, with subsequent influence on behavior and learning processes [2]. Simultaneously, the first 3 years of life seems to be critical for the assembly of the commensal ecosystem; during the second and third year the gut microbiota (GM) is subjected to important development, becoming more similar to the adult ecosystem [3].

Recent research suggests that the GM, a complex microbial ecosystem populating our gastro-intestinal tract, may be a key modulator of neurodevelopment through the microbiome-gut-brain axis [4, 5]. In rodents, experimental altering of the GM impacts anxiety- and depression-related behaviors in multiple well-established paradigms [6–9]. Cognitive effects have also been reported [10–12]. In humans, altered composition of the GM has been reported in children with psychiatric diseases such as autism [13–16] and attention-deficit/hyperactivity disorder [17], and has been linked to childhood temperament [18]. Besides postnatal environmental factors, prenatal influences like maternal factors [19], gestational exposures [20–23], and mode of delivery [24, 25], were all associated with the diversity and architecture of the infant’s GM during the first year of life.

Prenatal polycyclic aromatic hydrocarbon (PAH) exposure produced neurodevelopmental toxicity in both animal experiments [26–28] and human epidemiological studies [29–32]. Our previous functional studies showed that organic extracts from settled house dust could interfere with human thyroid-hormone metabolism by competitively antagonizing thyroid-hormone receptors [33]. More importantly, our previous study found that PAHs, especially high-molecular-weight PAHs in settled house dust were significantly correlated with children’s intellectual development and neurobehavioral problems. Additionally, PAH concentrations were positively correlated with severity of children’s neurobehavioral problems characterized by compulsivity and aggressivity [34]. These findings suggested a possible relationship between early indoor exposure to PAHs and children’s later neurobehavioral development, and consequently, intellectual and neurodevelopmental disorders. Our prior investigation showed that exposure to PAHs, represented by 10 hydroxyl metabolites, in toddlers at 12 months could influence their neurodevelopment.

Persistent organic pollutants (POPs) can change the diversity of the GM by activating aromatic hydrocarbon receptors (AhR), affect the host’s metabolic balance [35, 36], and further participate in the regulation of neurodevelopmental disorders-related behavior and physiological abnormalities [7]. As one of the POPs, PAHs are important ligands for AhR in the body. The load of PAHs in early childhood mainly comes from the digestive tract, and most of the PAHs, especially high molecular weight PAHs, are mainly excreted from the digestive tract through feces, not through the kidney [37]. Interestingly, our team’s findings show that PAHs in urban house dust that cause internalizing behavior problems are more likely to be of high molecular weight [34]. Therefore, we suspected a possible important role of the GM in the neurodevelopmental toxicity of PAHs.

Some psychiatric diseases like autism are extremely heterogeneous neurodevelopmental disorders and their onset can vary, with some children showing signs from early infancy and others exhibiting symptoms of regression at the age of 2–3 years. Notably, the first 3 years of life seems to be critical for the assembly of the commensal ecosystem. The fascinating observation of a critical period in which the microbiota could affect neurodevelopment and the existence of sensitive periods for pollutant-dependent refinement of sensory modalities (i.e., vision, hearing etc.), language acquisition, fear extinction and many others processes, suggests the appealing possibility of microbiome-driven stimuli involvement in fine-tuning neuronal circuits remodeling during early age. The present study is designed to explore the possible association between the composition of the GM and neurodevelopment in a sample of healthy 3-yr-old children. Simultaneously, early postnatal PAH exposure was measured to explore the possible influence on the GM.

## Methods

### Participants

The infants were selected from our prospective birth cohort established in 2014 [38, 39]. Pregnant women at the obstetrics clinic of the hospitals in Qingdao were invited to sign the informed-consent form for participation in this prospective cohort study. In order to avoid the possible influence of the mode of delivery, the type of feeding (breast or formula milk) and the use of antibiotics on the composition of the neonatal gut microbiota, additionally to meeting the criteria of eligibility of the cohort, the subjects included in the present study should be natural delivery, breastfeeding and have been free of antibiotic use in the preceding 30 days. In the end, only 38 had postnatal PAH exposure levels and underwent GM measurement. This study was approved by the Ethics Committee and Institutional Review Board of Qingdao Municipal Center for Disease Control and Prevention. The authors declare that all procedures contributing to this work comply with the ethical standards of the relevant national and institutional committees on human experimentation and with the Helsinki Declaration of 1975, as revised in 2008.

### 16S rRNA gene sequencing

Fresh fecal samples were collected from recruited subjects at 3 years of age and transported to Qingdao CDC laboratory with an ice packs within 2 h. All samples were then immediately frozen and stored at − 80 °C prior to analyses. The diversity and abundance of the GM were analyzed by 16S rRNA gene sequencing (V4-V5 region) with the Illumina MiSeq. DNA of the GM from stool was obtained by the QIAamp DNA Stool Mini Kit (Qiagen, Germantown, MD). Then, the DNA samples were sent to the TinyGene Company (Shanghai, China) for 16S rRNA gene-sequencing analysis.

### Postnatal PAH-exposure measurement

Morning first-void urine samples were collected for 5 consecutive days when the infants were able to urinate spontaneously (between 1.5 and 2 years old). The pooled sample of 5 consecutive days was used for the postnatal PAH measurement. Urinary hydroxyl PAH testing was performed following the method described by Guo et al. with minor changes [40] (Supplementary information). Our selected hydroxyl metabolites of PAHs included 10 exposure biomarkers which were: 1-, 2-hydroxynaphthalene; 2-hydroxyfluorene; 1-, 2-, 3-, 4-, 9-hydroxyphenanthrene; 1-hydroxypyrene; and 6-hydroxychrysene (Supplementary information).

### Measures of child neurodevelopment and behavior

The mothers of 2–3-yr-olds were asked to fill the Child Behavior Checklist (CBCL) to evaluate the children’s behavior [41]. The Chinese version of CBCL/2–3 has been translated and applied in China with a Cronbach α = 0.78, and validity coefficient = 0.73 [42]. The checklist contains six core syndromes: Anxious/depressed; Withdrawn; Sleep problems; Somatic complaints; Aggressive behavior; and Destructive behavior. In addition, there are two broadband syndromes: Internalizing and Externalizing.

The Gesell Development Inventory (GDI) was selected in the present study for the measures of child neurodevelopment. The GDI has been translated and standardized by the Beijing Children’s Health Care Institute, and consists of five behavioral domains: Adaptive; Gross motor; Fine motor; Language; and Personal social behaviors [43]. Each child was assigned a developmental quotient (DQ) in each of the five specific domains. The higher the DQ, the better the neurodevelopment. All subjects were tested by a professional doctor at the Department of Child Health Care at Huangdao Maternity and Child Care Hospital of Qingdao.

### Statistical analyses

All microbiome data were represented as proportions of total microbiome. Mean and standard deviation (SD) were used to describe the distribution of DQs and median and range were used for CBCL scores. Half of the limit of detection (LOD) was used for the OH-PAHs for the final statistical analyses. Due to small sample size and non-normal distributions, Spearman correlations examined the association between specific GM proportions and DQs and CBCL Scores. R-statistics (http://www.r-project.org/) was used for hierarchical clustering analysis and heatmaps of the Spearman correlation coefficient data to give insight into the structure of the data. Higher DQ and lower CBCL scores reflect better neurodevelopment. OH-PAHs were divided into high exposure (> 30 μg/g creatinine) and low exposure (≤ 30 μg/g creatinine) to explore the PAH influence on the GM. We further performed partial correlation analysis for the association between DQ and GM proportions with controls for OH-PAH and sex. All statistical analyses were performed using IBM SPSS 21.

## Results

### Characteristics of the study population

Of 38 children, 20 were boys (52.6%). Their average gestational age was 39 ± 1 weeks, and the average birth weight was 3604 ± 733 g. The mean DQs for all five behavior domains were all above 100. The medians for six CBCL core syndromes and two broadband syndromes were all less than 10. The geometric mean (95% confidence interval) for OH-PAH (μg/g creatinine) was 10.23 (6.44, 16.25) (Table 1).

**Table 1:** The general information, DQs and CBCL scores of the subjects (*n* = 38)

Variables	Subjects
Mother’s age(y)	
Mean ± SD	34 ± 3
Range	27 ~ 39
Gestational weeks(w)	
Mean ± SD	39.62 ± 1.01
Child’s birth weight(g), mean ± SD	
Mean ± SD	3604.17 ± 733.33
DQs, Mean ± SD	
Adaptive	102 ± 6
Gross motor	108 ± 7
Fine motor	108 ± 8
Language	103 ± 7
Personal social	106 ± 7
CBCL syndromes, median (range)	(*n* = 25)
Anxious/depressed	3 (0, 13)
Withdrawn	3 (0, 14)
Sleep problems	2 (0, 8)
Somatic complaints	3 (0, 16)
Aggressive	4 (0, 19)
Destructive	3 (0, 11)
Internalizing	5 (0, 23)
Externalizing	7 (0, 26)
Total CBCL score	24 (4, 95)
OH-PAH/Cr (μg/g Cr)	
GM (95% CI)	10.23 (6.44,16.25)

*SD* standard deviation, *Cr* creatinine, *GM* geometric mean, *CI* confidence interval

### Association between GM and neurodevelopment

**Fig. 1:**
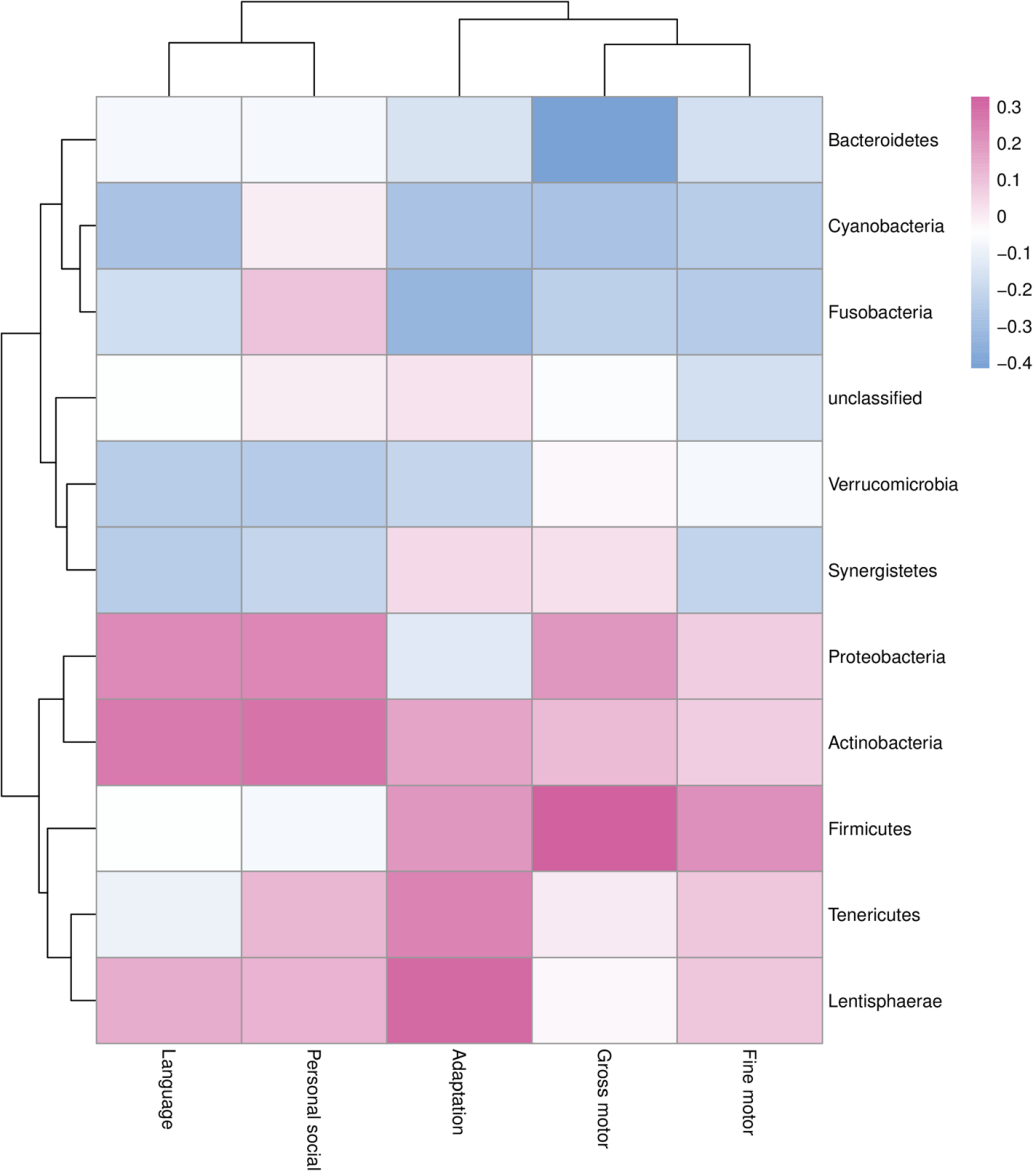
Heatmap of the Spearman correlation coefficients between scores of five GDI behavior domains and abundance of 11 GM phyla. Color-coded with blue for negative lower coefficients and red for positive higher coefficients. Dendrograms present clustering of GM phyla (rows) and GDI behavior domains (columns), which is based on hierarchical clustering with Euclidean distance metric and average linkage

The most abundant GM phyla were *Bacteroidetes* and *Firmicutes* with mean ± SD relative abundance 0.6862 ± 0.0940 and 0.2420 ± 0.0805, respectively (Figure S1). The phyla of *Firmicutes*, *Actinobacteria*, *Proteobacteria*, *Tenericutes*, and *Lentisphaerae* were positively correlated with most domain behavior scores of the GDI. *Firmicutes* showed a significant correlation with Gross motor behavior score (*r* = 0.327, *P* < 0.05). *Bacteroidetes*, *Cyanobacteria*, and *Fusobacteria* showed negative correlations with most domain behaviors, of which *Bacteroidetes* showed a significant correlation with the Gross motor behavior score (*r* = − 0.416, *P* < 0.01) and *Fusobacteria* with the Adaptive behavior score (*r* = − 0.334, *P* < 0.05) (Fig. 1).

Consistent with the phylum level, the lower taxa *Bacteroidia*, *Bacteroidales*, *Myroides*, *Bacteroides stercoris*, *Prevotella buccae*, *Myroides sp. A21* and *Myroides odoratus* of the *Bacteroidetes* showed significant negative correlations with some domain behaviors of the GDI (Figures S2-S6). For the *Firmicutes* phylum, the *Ruminococcaceae*, *Christensenellaceae*, *Ruminococcus*, *Ruminiclostridium*, *Anaerotruncus*, *Hungatella*, *Ruminococcus sp N15MGS57*, *Bacterium enrichment culture clone Ecwsrb027*, *Clostridium lavalense*, *Uncultured prokaryote*, *Ruminococcaceae bacterium, Uncultured Veillonellaceae bacterium*, *Bacterium enrichment culture clone Ecwsrb027*, *Uncultured Veillonellaceae bacterium*, *Eubacterium ramulus*, *Bacterium YE57*, *Christensenella minuta* and *Streptococcus mutans* showed significant positive correlations with some domains of the GDI (Figures S2-S6).

**Fig. 2:**
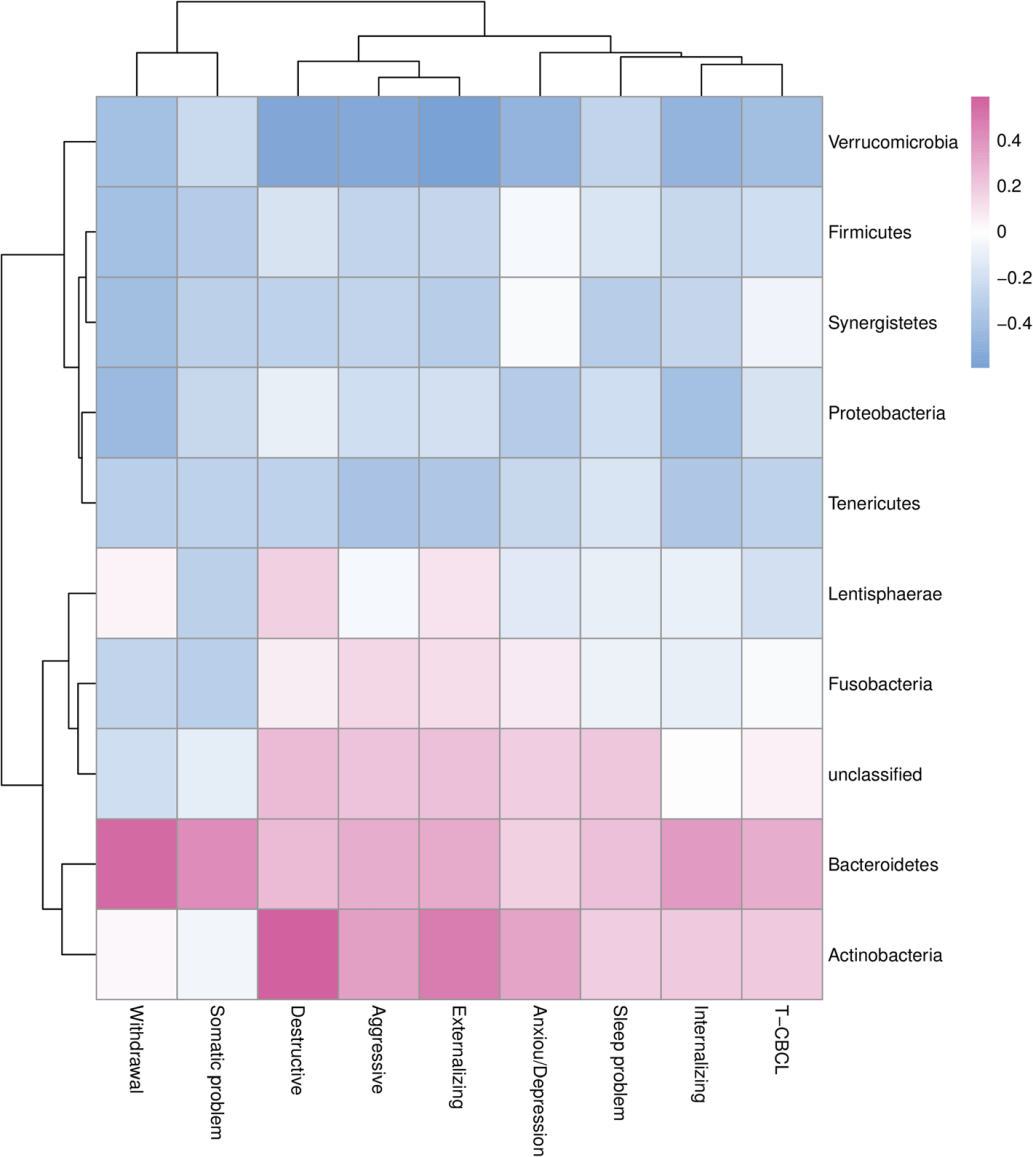
Heatmap of the Spearman correlation coefficients between scores of six core syndromes, two broadband syndromes, and total Score of CBCL and abundance of 11 GM phyla. Color-coded with blue for negative lower coefficients and red for positive higher coefficients. Dendrograms present clustering of GM phyla (rows) and CBCL syndromes (columns), which is based on hierarchical clustering with Euclidean distance metric and average linkage

Contrary to the positive direction of the DQs, the lower the CBCL score the better the neurodevelopment. For the phyla *Bacteroidetes*, *Actinobacteria*, and *Fusobacteria*, positive significant correlations were between *Bacteroidetes* and Withdrawn syndromes (*r* = 0.551, *P* < 0.01), and Somatic complaints (*r* = 0.413, *P* < 0.05). For *Actinobacteria,* positive correlations were found with Destructive behavior (*r* = 0.589, *P* < 0.01) and Externalizing behavior (*r* = 0.471, *P* < 0.05). The phyla *Firmicutes*, *Verrucomicrobia*, Synergistetes, *Proteobacteria* and *Tenericules* showed significant negative correlations on CBCL between *Proteobacteria* and Withdrawn syndromes (*r* = − 0.435, *P* < 0.05), *Verrucomicrobia* and Anxious/depressed (*r* = − 0.476, *P* < 0.05), Aggressive (*r* = − 0.542, *P* < 0.05), Destructive (*r* = − 0.552, *P* < 0.01), Internalizing (*r* = − 0.471, *P* < 0.05), Externalizing behaviors (*r* = − 0.598, *P* < 0.01), and the total CBCL scores (*r* = − 0.410, *P* < 0.05) (Fig. 2).

Consistent with the phylum level, the *Bacteroidia*, *Bacteroidales*, *Bacteroides*, *Bacteroides vulgatus*, *Bacteroides intestinalis*, *Bacteroides uniformis*, and *Bacteroides caccae* within *Bacteroidetes* phylum all showed significant positive correlation with most CBCL behavior syndromes (Figures S7-S11). Within *Firmicutes,* the *Anaerostipes*, *Oscillospira*, *Lactobacillus*, *Caproiciproducens, Selenomonas*, *Ezakiella*, *Moryella*, *Acetitomaculum*, *gut metagenom*, and *uncultured rumen bacterium* all showed negative correlations with most CBCL behavior syndromes. As with most lower taxa of the *Firmicutes* phylum, within *Verrucomicrobia*, all detected lower taxa showed negative correlations with all CBCL behavior syndromes.

Despite showing mostly consistency, the lower taxa within the phylum still showed some variance. For example, within the phylum *Bacteroidetes*, the family *Bacteroidaceae* showed the same associations as the order *Bacteroidales*, but the family *Prevotellaceae* showed negative correlations with almost all CBCL behavior syndromes (Figure S9).

### Influence of the PAH exposure on GM and neurodevelopment

**Fig. 3:**
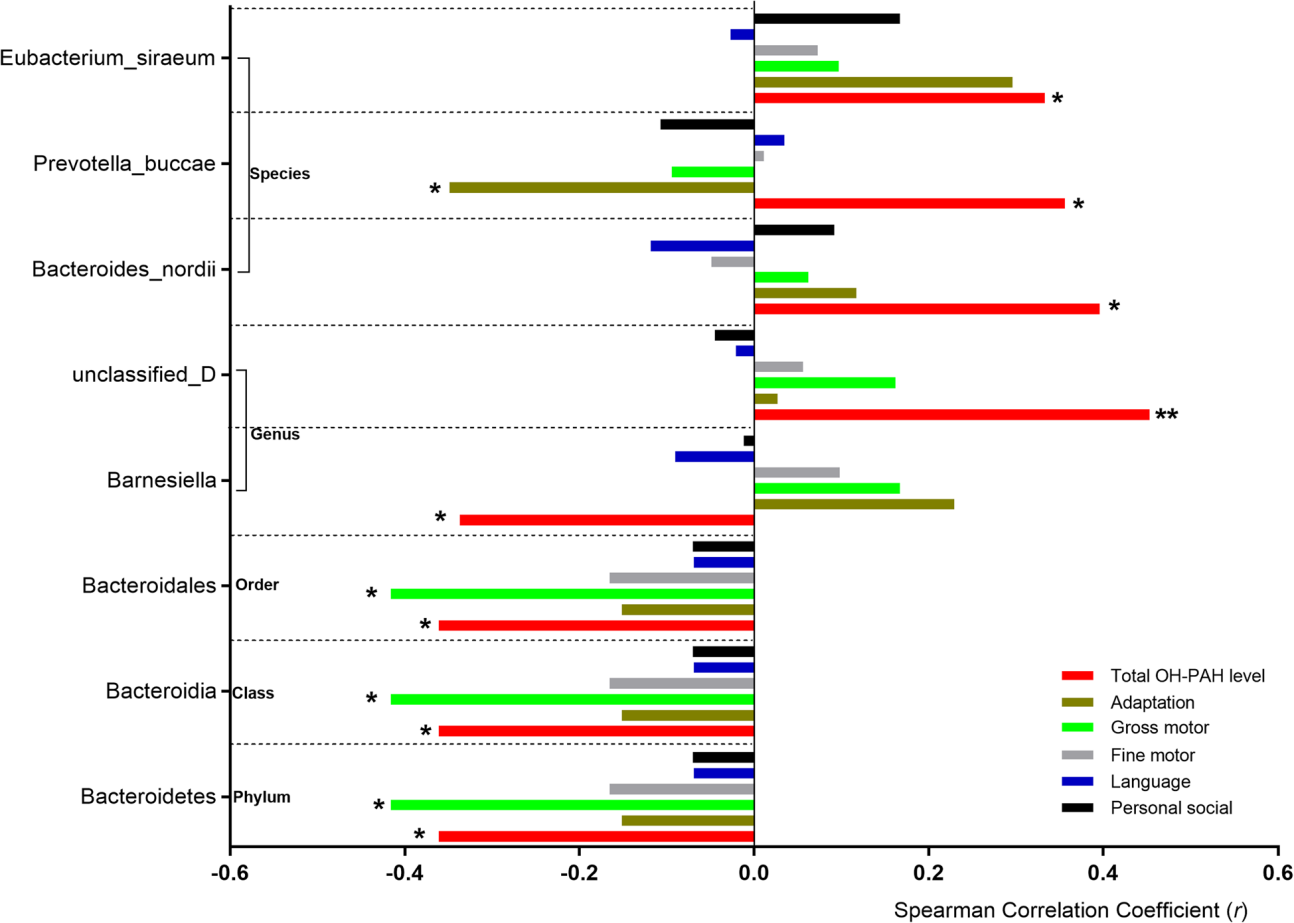
The Spearman correlation coefficients between the abundance of the GM and OH-PAHs (red) and scores of five GDI behavior domains. *P < 0.05; **P < 0.01

**Fig. 4:**
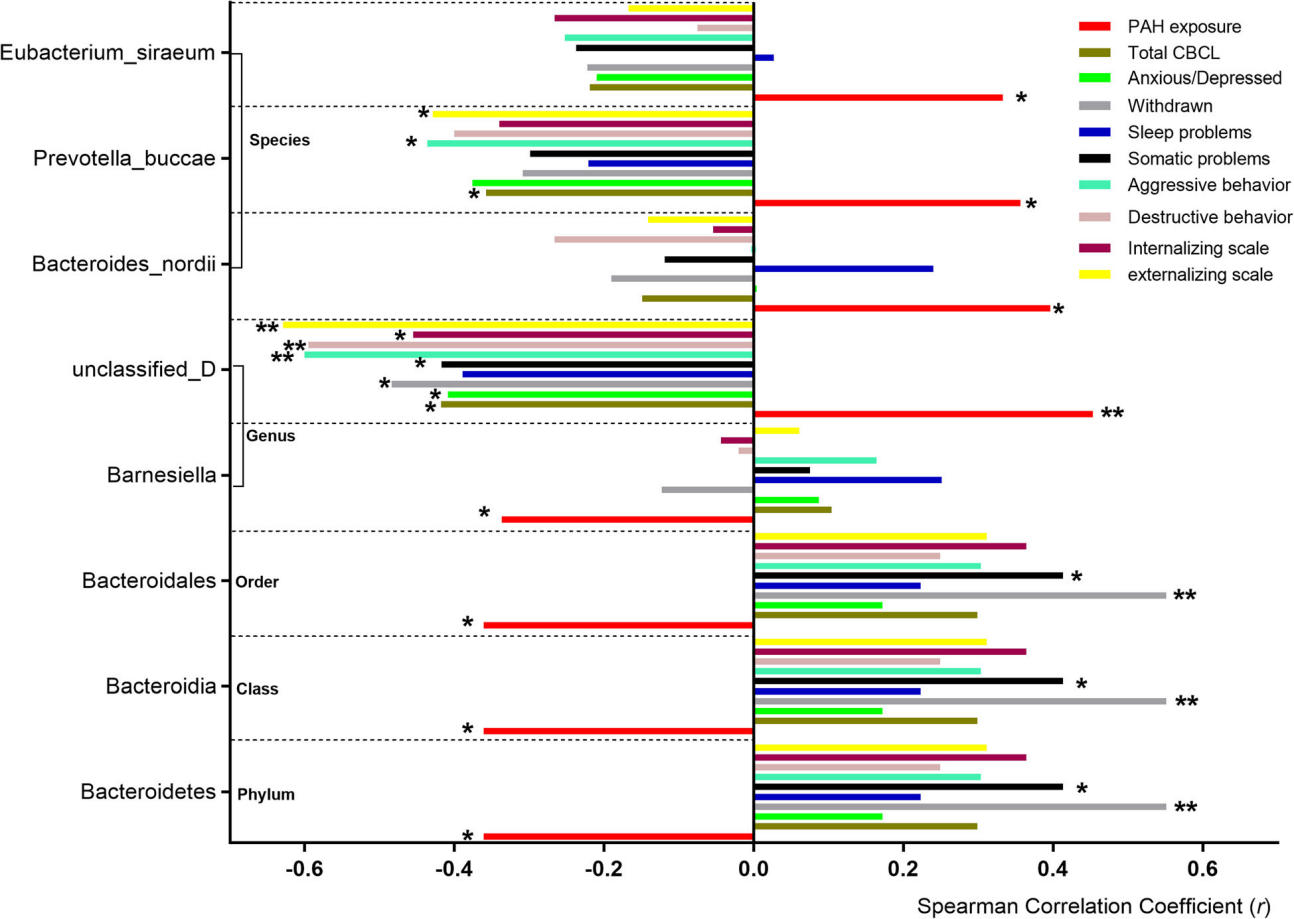
The Spearman correlation coefficients between the abundance of the GM and OH-PAHs (red) and scores of the CBCL behavior syndromes. *P < 0.05; **P < 0.01

We explored the influence of the PAH exposure on the relative abundance of the GMs. Within the *Bacteroidetes* phylum, the microbiota showed negative association with the OH-PAH levels, of which the *Bacteroidia* class, the *Bacteroidales* order, and the *Barnesiella* genus showed statistical significance (Fig. 3). The *Firmicutes* phylum seemed to show positive association although no statistical significance was found. After we divided the OH-PAH levels into high- and low-exposure groups, of the lower taxa, the *Negativicutes* class, the *Selenomonadales* and *Bacillales* order, the *Lactobacillaceae* family and the *Lactobacillus* genus all showed significantly higher relative abundance in high-exposure group, whereas the *Clostridium perfringens*, the *Clostridium Colinum*, and the *Clostridium sp 7 3 54FAA* species all showed lower relative abundance (Fig. 4). Additionally, *Roseburia*, *Ruminococcus*, *Blautia*, *Dialister*, *Coprococcus*, *Megasphaera*, *Eubacterium*, and *Anaerostipes* all showed negative association with the postnatal PAHs exposure levels (Figures S10-S11).

**Fig. 5:**
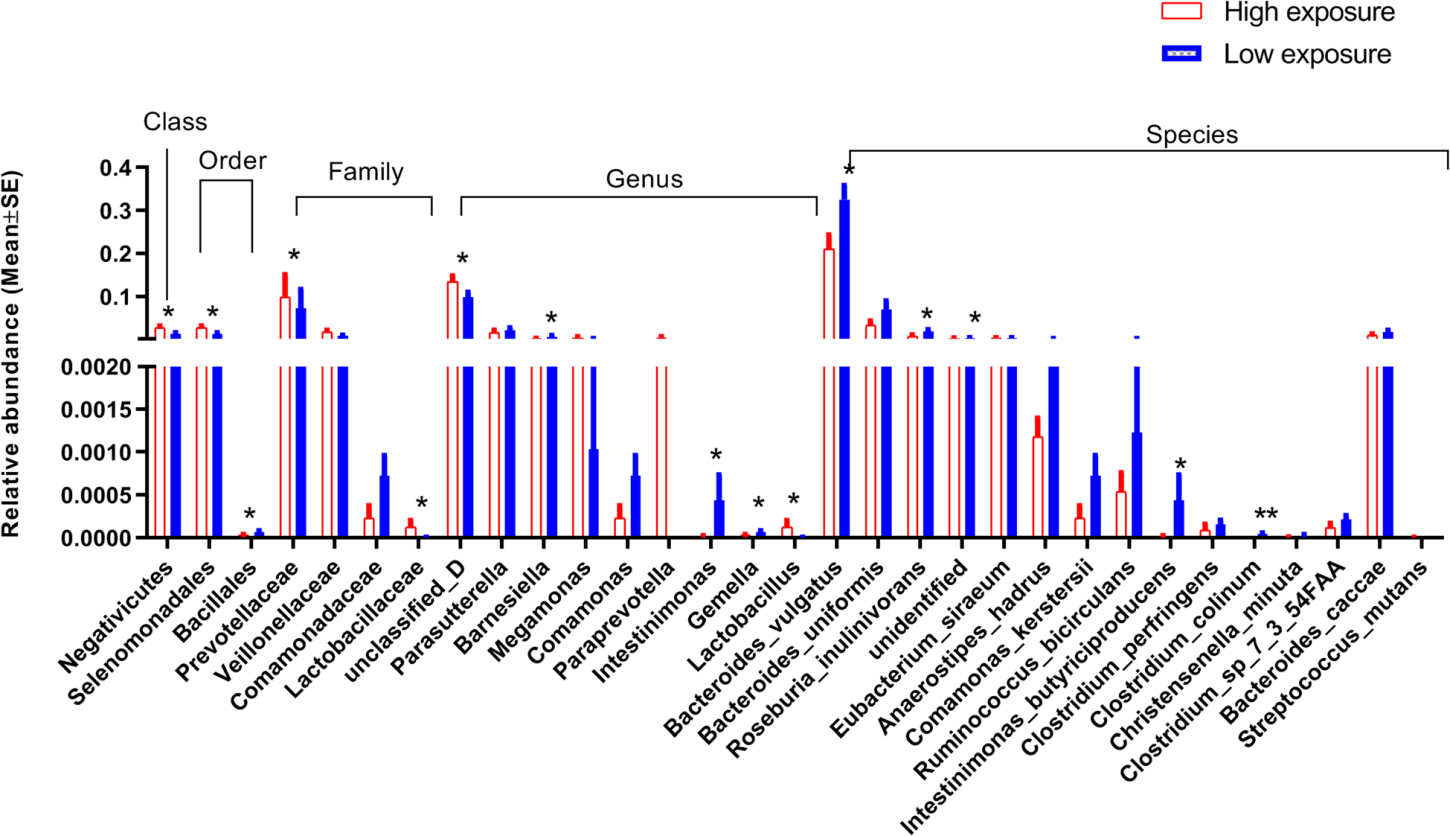
The differences of the relative abundance of the GM between high (> 30 μg/g creatinine) and low (≤30 μg/g creatinine) PAH exposure. (Only the GMs with *P* < 0.1 were showed) *P < 0.05; **P < 0.01. SE: standard error

Although the *Bacteroidetes* phylum, the *Bacteroidia* class, and the *Bacteroidales* order all showed negative association with OH-PAH levels and all GDI domain behaviors (Fig. 3), and positive association with all CBCL behavior syndromes (Fig. 4), the *Bacteroides nordii*, *Prevotella buccae* and *Eubacterium siraeum* species all showed positive association with OH-PAH levels, but indicated negative correlation with most CBCL behavior syndromes (Fig. 4). It is interesting that the unidentified bacteria at the genus level showed significant positive correlation (*r* = 0.453, *P* < 0.001) with OH-PAH levels and significant negative correlation with most CBCL behavior syndromes (Fig. 5).

**Fig. 6:**
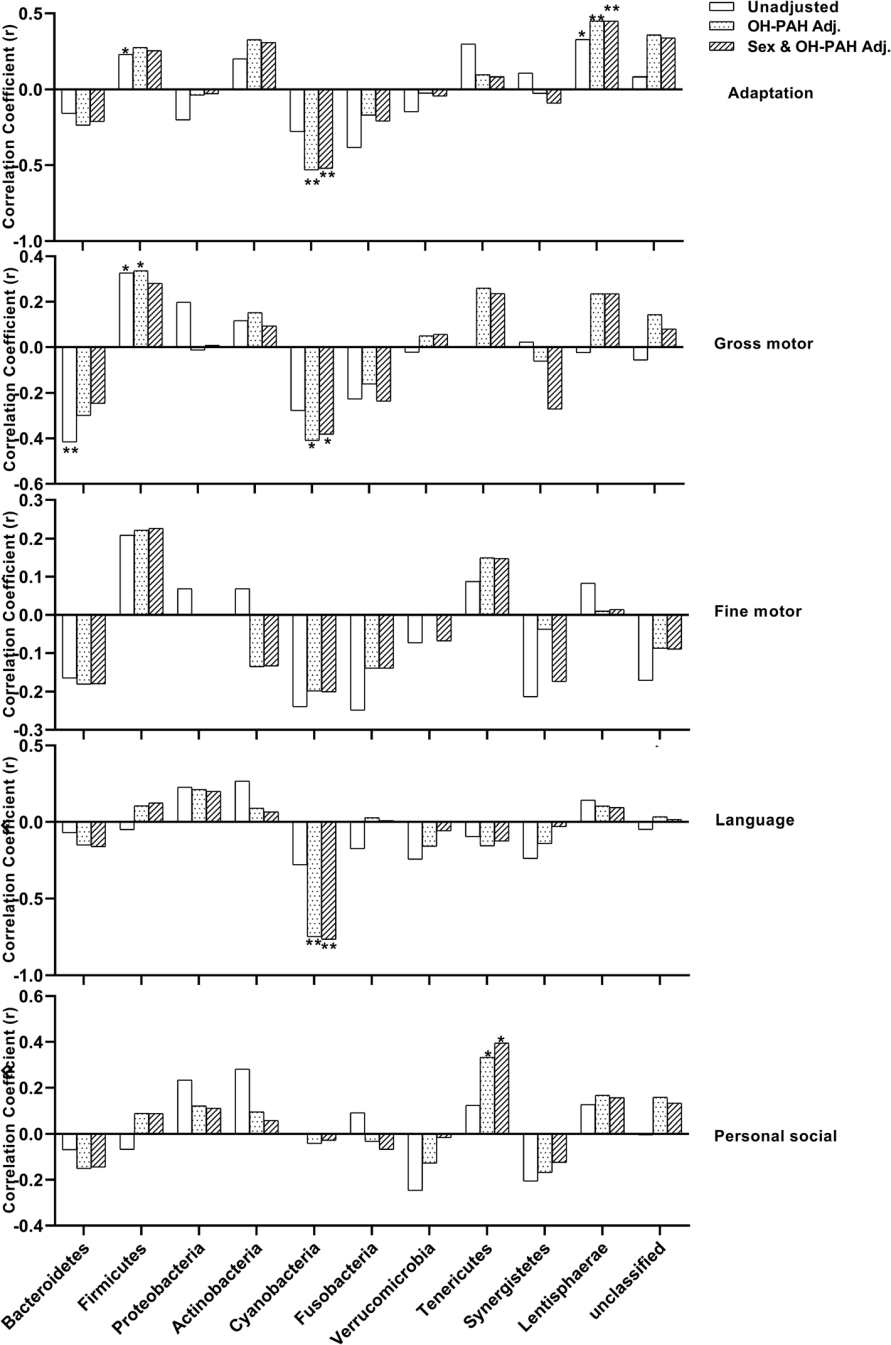
The Spearman correlation coefficients between scores of five GDI behavior domains and abundance of 11 GM phyla with or without adjustment for hydroxyl PAHs and sex

When we further adjusted for OH-PAHs to explore the PAH exposure influence on the association between the GM and GDI, the significant correlation between *Bacteroidetes* and gross motor behavior disappeared (Fig. 6). After adjusting for OH-PAHs, the phylum *Cyanobacteria* showed a significant negative association with Adaptation (*r* = − 0.532, *P* < 0.01), Gross motor (*r* = − 0.410, *P* < 0.05), and Language (*r* = − 0.749, *P* < 0.01) domain behavior; the phylum *Tenericutes* showed a significant positive association with Personal social domain behavior (*r* = 0.395, *P* < 0.05). These associations did not change much after adjustment for sex.

## Discussion

The composition of the GM can have a profound impact on human health. Accumulating evidence has suggested that environmental pollution may be associated with several pathologies [44]. In the present study, the GM composition in 3-year-old children was measured and the internal PAH concentration was analyzed to explore the possible influence on the GM and related neurobehavioral development.

A recent study investigated whether the composition of the GM at 1 year of age was associated with cognitive outcomes [45]. The cognitive outcomes were measured by the Mullen Scales of Early Learning in 89 infants at 1 year of age. The global and regional brain volumes were obtained with structural magnetic resonance imaging at 1 and 2 years. The neuroimaging data suggested that GM might have minimal effects on regional brain volumes at 1 and 2 years of age. But Mullen scores at 2 years of age differed significantly between clusters of GM. That was the first study to demonstrate associations between the GM and cognition in human infants. However, GM was assessed at 1 year of age when intake of solid foods varies significantly, potentially resulting in rapid shifts of GM. By contrast, in the present study the GM was assessed at 3 years of age, and *Bacteroidetes* and *Firmicutes* were the top two dominant phyla. This is consistent with those reports of children in other studies [46], and in adults [47].

Multiple connections exist between the gastrointestinal tract and the brain, including immune, metabolic, circulatory, and neuronal pathways [4, 48]. Commensal GM is known to regulate various aspects of each, potentially leading to changes in host behavior. The GM can alter levels of pro-inflammatory cytokines produced by macrophages and T cells in the gastrointestinal tract [49], and microglia in the brain [50]. Microglia have been linked to behavioral changes associated with neurodegenerative disorders and may thereby be an indirect mechanism through which bacteria influence host behavior [51]. We used two behavior scales, GDI and CBCL, to evaluate infant neurodevelopment. Higher DQs of the GDI means better development. Conversely, higher scores of the CBCLs means poorer development. Positive and negative results of the two scales indicate a better understanding of the association between the GM and the neurodevelopment.

From both GDI and CBCL scales, our results indicated that higher proportions of *Firmicutes* and *Verrucomicrobia* at the phylum level may in some way be beneficial or protective for the development of neural system, whereas higher proportions of *Bacteroidetes* may be less beneficial, or possibly deleterious. Consistent with our results are reports of negative associations between proportion of *Bacteroidetes* and cognitive impairment in persons with severe liver dysfunction [52] and positive associations between *Verrucomicrobium* and learning and memory performance in the microbiome depletion/transplantation mouse model [53].

At the lower taxa of the phylum level, the GM composition shifts seem sophisticated. Some orders of the *Bacteroidetes* and *Firmicutes* showed consistent association; this was also found in a mouse model with diet-caused changes in the GM and cognitive flexibility [54]. In that study, both increased *Clostridiales* order of the *Firmicutes* phylum and lower percentages of *Bacteroidales* were found to be related to searching more for the old platform position than the normal animals.

No consensus has emerged from previous human studies of mental disorders and GM concerning which bacterial taxa are most relevant. However, species from the *Lactobacillus* have been used as a therapeutic intervention to suppress depressive-like behaviors in animal models [55, 56]. In the present study, the *Lactobacillus* genus, and *Lactobacillus delbrueckii* species were all negatively correlated with CBCL behaviors. The genera of the *Eggerthella*, *Halomonas*, and *Turicibacter* indicated positive association with the CBCL behaviors, which is consistent with the changes in a rat model of major depression [57]. Our results showed that *Bacteroides coprocola* species correlated negatively and *Bacteroides uniformis* correlated positively with the CBCL behavior syndromes; this is consistent with results reported in Attention-deficit/hyperactivity disorder (ADHD) [58]. The beneficial effects of the *Akkermansia* and *Lactobacillus* genera found in the present study were consistent with those reported in a recently published meta-analysis for association between the GM and Autism Spectrum Disorder [59].

PAHs are considered to be high-priority environmental contaminants not only because of their toxic, carcinogenic, and putative estrogenic or antiestrogenic properties in humans [60, 61] but also because of neurodevelopmental toxicity in children [29–32]. Ingested PAHs reach the intestine enterocytes and liver hepatocytes and act as AhR ligands. Moreover, microbiota in the colon can catalyze the conversion of PAHs to estrogen. This bioactivation could be an underlying mechanism of PAH toxicity [61].

Oral exposure to Benzo(a) pyrene (BaP)-inducing gut microbial shifts in a murine model has been reported [62]. Contrary to our results, that study reported that after 28 days of subchronic oral exposure to BaP, there was an increase in the pro-inflammatory bacterial taxa belonging to the families *Alcaligenaceae*, *Bacteroideceae*, *Erysipelotrichaceae*, *Paraprevotellaceae*, *Porphyromonadaceae* and *Turicibacter*. In contrast, populations of the anti-inflammatory bacterial taxa including *Lactobacillaceae*, *Lachnospiraceae*, *Ruminococcaceae*, and *Verrucomicrobiaceae* were reduced [62]. Our results seemed more concordant with the transient protective effects against the pro-inflammatory proprieties of BaP at the beginning of the experiment. After the first BaP oral administration, an increase in relative abundance of beneficial GM *Akkermansia muciniphila* was observed. This apparent trend was also found in our results although with no statistically significant difference.

Although we did not confirm our hypothesis at the high taxa, we did find negative associations of PAH levels with the *Roseburia*, *Ruminococcus*, *Blautia*, *Dialister*, *Coprococcus*, *Megasphaera*, *Eubacerium* and *Anaerostipes*, which were related to the gut production of the microbial metabolites propionate, acetate, or butyrate. As the members of the short chain fatty acids, propionate, acetate, and butyrate were depleted in the patients with major depressive disorder (MDD) through potential mechanisms of epigenetics (HDACi and DNA methylation modulator) or receptors like GPR43 and GPR41 [63].

Our results seemed more consistent with another two AhR modulators, 2, 3, 7, 8-tetrachlorodibenzo-p-dioxin (TCDD) [64], and polychlorinated biphenyls (PCBs) [65], which have been found potentially to shape the gut microbiome composition in mice. TCDD or PCB administration dramatically increased the abundance of *Firmicutes* and decreased the abundance of *Bacteroidetes* in mice. At the family level, the levels of *Lactobacillaceae* and *Desulfovibrionaceae* significantly increased and the levels of *Prevotellaceae* decreased in the feces after TCDD exposure.

Our results also indicated that the *Lactobacillus* genus of the *Lactobacillaceae* family positively associated with PAH levels. The *Lactobacillus* genus has been shown to produce acetylcholine (Ach), another kind of microbial metabolite [4, 63, 66]. Ach is essential for memory-trace formation and long-term memory, and Ach level reflects acetylcholine esterase (AchE) activity. AchE regulates signal transmission among cholinergic brain neurons by degrading Ach. AchE activity has been reported to decrease significantly in regions with high air concentrations of PAHs [67]. Serum Ach increase was also reported in a population of Chinese coke-oven workers with a correlation to neurobehavioral function [68]. Interestingly, tobacco smokers were found to be very resistant to ulcerative colitis [69], and the *Lactobacillus reuteri* recolonization was sufficient to recover metabolic function and behavioral impairment in chronically depressed mice [55, 56, 70]. Therefore, the relationship between the GM and the PAH levels in the present study seems to show that the recruitment of some GM can counteract the negative effects of the PAHs, thereby contributing to maintaining the gut ecosystem stability via a resistance phenomenon.

Another interesting finding of the present study was that PAH exposure seemed to weaken the negative association between the Cyanobacteria phylum and neurodevelopment. Cyanobacteria in the gut microbiota may produce the neurotoxins β-N-methylamino-L-alanine, saxitoxin, and anatoxin-α, which are considered to be related to the development of some neurological diseases like Alzheimer’s disease [71, 72] and to aging [71]. The exact nature of the counteracting effects of the PAHs on the Cyanobacteria needs further investigation.

In the present study, we only recruited 38 infants, of which only 25 had CBCL scores measured. Because the sample size of CBCLs was small and many partial correlation coefficients were missed, the partial correlation analysis was not performed for GM and CBCL scores. The small sample size may be our major limitation. A small sample size might cause low statistical power and a failure of detecting a significant difference. Therefore, we did not perform multivariate analysis and the correlation coefficients were presented with heatmap and hierarchical clustering. Although none of the previously reported studies calculated a sample size based on a predefined hypothesis [73], just picking the sample size might prevent us from defining an a priori probability of being wrong due to false negatives and false positives. Although our infants were recruited from a birth cohort, the present study was only an explorative design. The absence of evidence on the association between microbiome and the neurodevelopmental symptoms does not mean evidence of a negative association. Next, we only analyzed the composition of the GM. However, no substantial shifts in the bacterial communities of human fecal microbiota were observed in vitro [74], but acute BaP exposure greatly altered gut bacterial activity, regarding the volatolome (synthesis of volatile organic compounds) and the metatranscriptome, in a dose-dependent manner. This implies that BaP can modify the gut microbial ecosystem through GM activity. Finally, GM and neurodevelopmental testing were performed at the same age, which means the nature of the cross section and unsure of the causal link between GM and neurodevelopment.

Further high-quality studies are needed to better understand this topic. The microbiome at birth, and subsequently at regular intervals until adulthood, should be characterized. The possible presence of subjects with a diagnosis of neurodevelopmental disorders should then be assessed. High-quality and comprehensive measurement methods are needed to evaluate young children’s PAH exposure. Simultaneously, large differences between the dietary factors and physical activity should be included in the analyses.

## Conclusions

The present study suggests that the composition of the GM is associated with neurodevelopment, and this can and should be assessed in children by 3 years of age. Simultaneously, this association was somewhat consistent with those results from the animal disease model studies or from patients with neurodevelopmental disorders. PAHs seemed to change the relative abundance of some taxa (some deleted and some recruited) to counteract the negative effects of the PAHs. To better clarify the potential role of microbiome in the association between the environmental PAH exposure and the neurodevelopmental disorders, further accurate and reliable evidence is needed.

## Supplementary Information

**Additional file 1.**

## Data Availability

The datasets used and analysed during the current study are available from the corresponding author on reasonable request.

## Abbreviations

PAHs: Polycyclic aromatic hydrocarbons
GM: Gut microbiota
GDI: Gesell Development Inventory
POPs: Persistent organic pollutants
AhR: Aromatic hydrocarbon receptors
BaP: Benzo [a]pyrene
CBCL: Child Behavior Checklist
DQ: Developmental quotient
LOD: Limit of the detection
ADHD: Attention-deficit/hyperactivity disorder
MDD: Major depressive disorder
PCBs: Polychlorinated biphenyls
AchE: Ach level reflects acetylcholine esterase
UC: Ulcerative colitis
