# Supplementary material for: Preliminary evidence for an influence of exposure to polycyclic aromatic hydrocarbons on the composition of the gut microbiota and neurodevelopment in three-year-old healthy children
Source: BMC Pediatr. 2021 Feb 17;21:86. doi: 10.1186/s12887-021-02539-w (PMC7888120; doi:10.1186/s12887-021-02539-w)
Supplement: Supplementary file 1 — Additional file 1. [file 12887_2021_2539_MOESM1_ESM.docx]

**Supplement:**

***Umbilical cord blood and urine collection***

For each infant, 10 mL umbilical cord blood was collected using a 10 mL heparin anticoagulant vacuum tube. Samples were frozen thereafter and sent to the Qingdao CDC to reserve at -70℃.

For morning urine samples, parents of participants brought home three 15-mL polypropylene centrifuge tubes to collect 15 mL of their child’s morning urine each time for three days in a row. Samples were frozen immediately after collection and sent back to their hospital at birth, and then were reserved out of the sun at -20℃. These samples were finally sent to the Qingdao CDC and kept at -70℃ for later analysis.

***Urinary hydroxy PAHs testing***

We planned to collect urine samples in infants at the age of one year. But most infants at the age of one year were not able to urinate spontaneously. Thus, we collected the morning first-void urine samples when the infants were able to urinate spontaneously between 1 and a half and 2 years old. The caregivers took back the bottles and collected the urine samples in a consecutive 5 days. After mixing the urine samples collected continuously, we accurately removed 10 mL of the mixed sample to a blowing bottle, and then added 30 μL hydrolysate of β-glucoaldosidase and aryl sulfatase. Later we placed the fully blended mixture to a 37℃ thermostatic incubator to stay overnight. Afterwards, we collected these hydrolyzed samples to enrich and purify in a C18 SPE column (6 mL/500 mg). The C18 columns were first activated by a 5 mL of acetonitrile and a 10 mL of water. Samples passed columns at a speed of 1mL/min. After samples passed, these C18 columns were cleaned with a 3 mL of water and drained under negative pressure. Then, they were eluted with an 8 mL of formic acid-acetonitrile (0.3%, v/v). Eluents were then dried and condensed to less than 1 mL by nitrogen under 35℃, and diluted to 1 mL under mobile phase with the initial ratio. They were ready for LC-MS/MS testing after being filtered by a 0.22μm of membrane.

An Acquilty UPLC BEH-C18（100 mm×2.1 mm, 1.7µm, Milford, USA）was equipped. The flow rate was 0.5 mL/min. Mobile phases A and B were water and acetonitrile, respectively. The gradient of mobile phase A was programmed as follows: 60% A at 0.0–0.2 min, then the percentage of A was decreased linearly to 5% between 0.2–3.0 min; 5% A between 3.0–4.0 min, then the percentage of A was increased to 60% from 4.0–4.1 min, and 60% A between 4.1-6.0 min. The temperature of the chromatography column was maintained at 40 ℃ in a column oven. The injection volume was 10 µLm in a partial loop using a needle overfill injection mode.

The MS/MS detector was equipped with an ESI interface and operated in positive ionization mode. The quantitative analyses were carried out in multiple reaction monitoring (MRM) mode. The conditions for the MS/MS detector were set as follows: capillary voltage, 3.00kV; ion source temperature, 150 ^o^C; desolvation gas flow, 1000 L/hr; desolvation gas temperature, 500 ^o^C; and cone gas flow, 50L/hr. Other parameters like quantity ion pairs (m/z), cone voltage and collision energy were listed in STable1.

**Table S1** The quantity ion pairs, cone voltage and collision energy of ten hydroxyl metabolites of PAHs.

| Metabolites | Retention time (min) | Quantity ion pairs (m/z) | Cone voltage/V | Collision energy/eV |
| --- | --- | --- | --- | --- |
| 1-OHNap | 1.45 | 143/119 | 25 | 32 |
| 2-OHNap | 1.60 | 143/119 | 25 | 32 |
| 2-OHFlu | 1.98 | 181/153 | 21 | 41 |
| 2(3)-OHPhe | 2.12 | 193/165 | 28 | 35 |
| 1(9)-OHPhe | 2.25 | 193/165 | 28 | 35 |
| 4-OHPhe | 2.35 | 193/165 | 28 | 35 |
| 1-OHP | 2.53 | 217/189 | 33 | 30 |
| 6-OHChr | 2.78 | 243/215 | 32 | 40 |

**Table S2** Detection rate and accuracy test.

| Metabolites | Detection limit (µg/L) | Standard concentration  (µg/L) | Average measured  concentration (µg/L) | Average recovery rate  (%) |
| --- | --- | --- | --- | --- |
| 1-OHNap | 0.0056 | 5.00 | 4.16 | 83.2 |
| 2-OHNap | 0.0076 | 5.00 | 4.32 | 86.4 |
| 2-OHFlu | 0.0270 | 5.00 | 4.07 | 81.4 |
| 2(3)-OHPhe | 0.0088 | 10.00 | 11.36 | 114 |
| 1(9)-OHPhe | 0.0053 | 10.00 | 11.10 | 111 |
| 4-OHPhe | 0.0080 | 5.00 | 5.04 | 100 |
| 1-OHP | 0.0120 | 5.00 | 3.80 | 76.0 |
| 6-OHChr | 0.0010 | 5.00 | 3.60 | 72.0 |


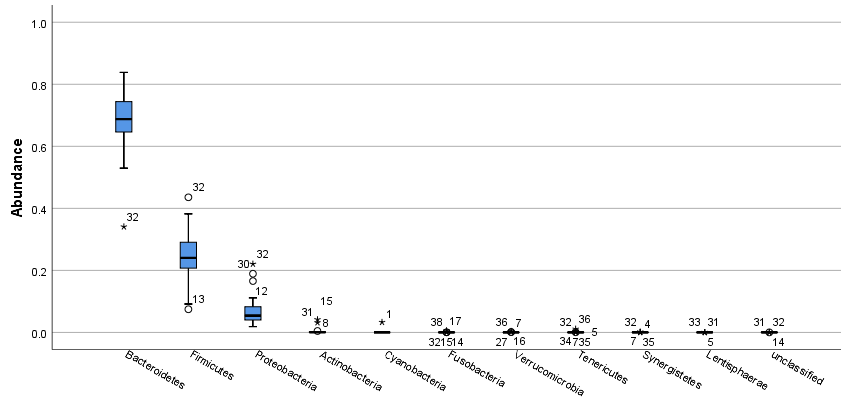


**Fig. S1** The distribution of the relative abundance of the 11 GM phyla.


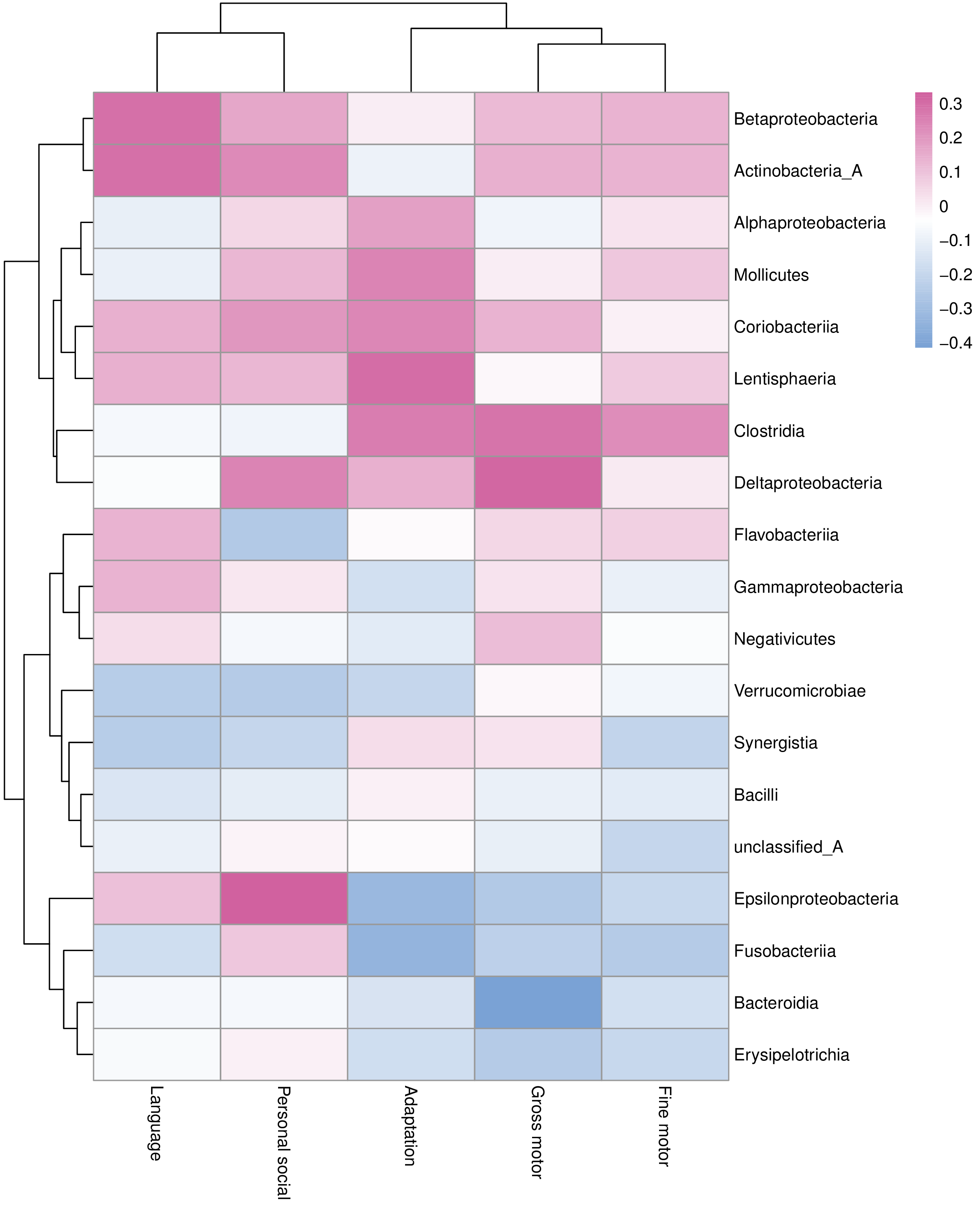


**Fig. S2** Heatmap of the Spearman correlation coefficients between scores of five GDI behavior domain and abundance of 19 GM classes. Color-coded with blue for minus lower coefficient and red for positive higher coefficient. Dendrograms present clustering of GM classes (rows) and GDI behavior domains (columns) which is based on hierarchical clustering with Euclidean distance metric and average linkage.


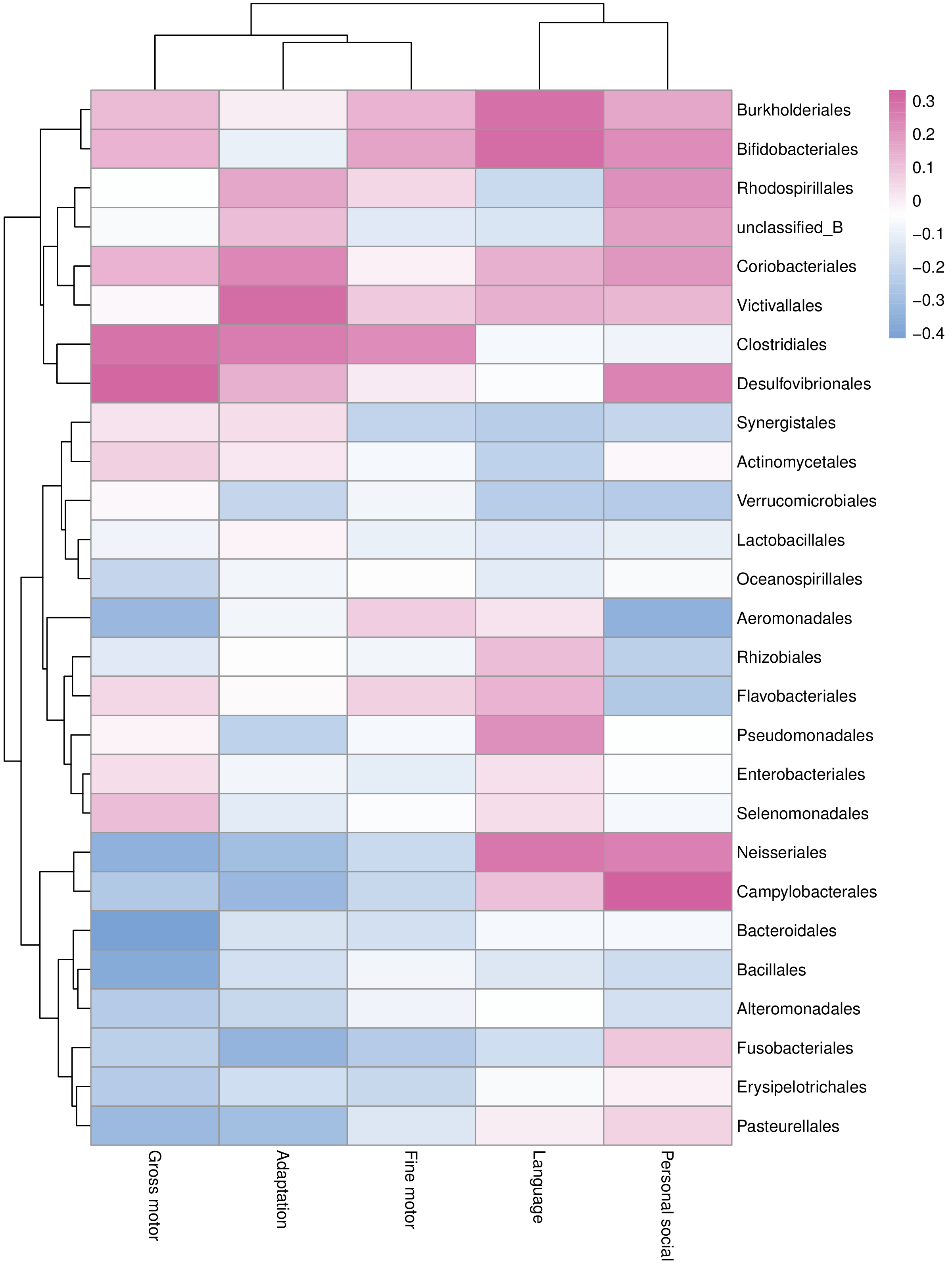


**Fig. S3** Heatmap of the Spearman correlation coefficients between scores of five GDI behavior domain and abundance of 27 GM orders. Color-coded with blue for minus lower coefficient and red for positive higher coefficient. Dendrograms present clustering of GM orders (rows) and GDI behavior domains (columns) which is based on hierarchical clustering with Euclidean distance metric and average linkage.


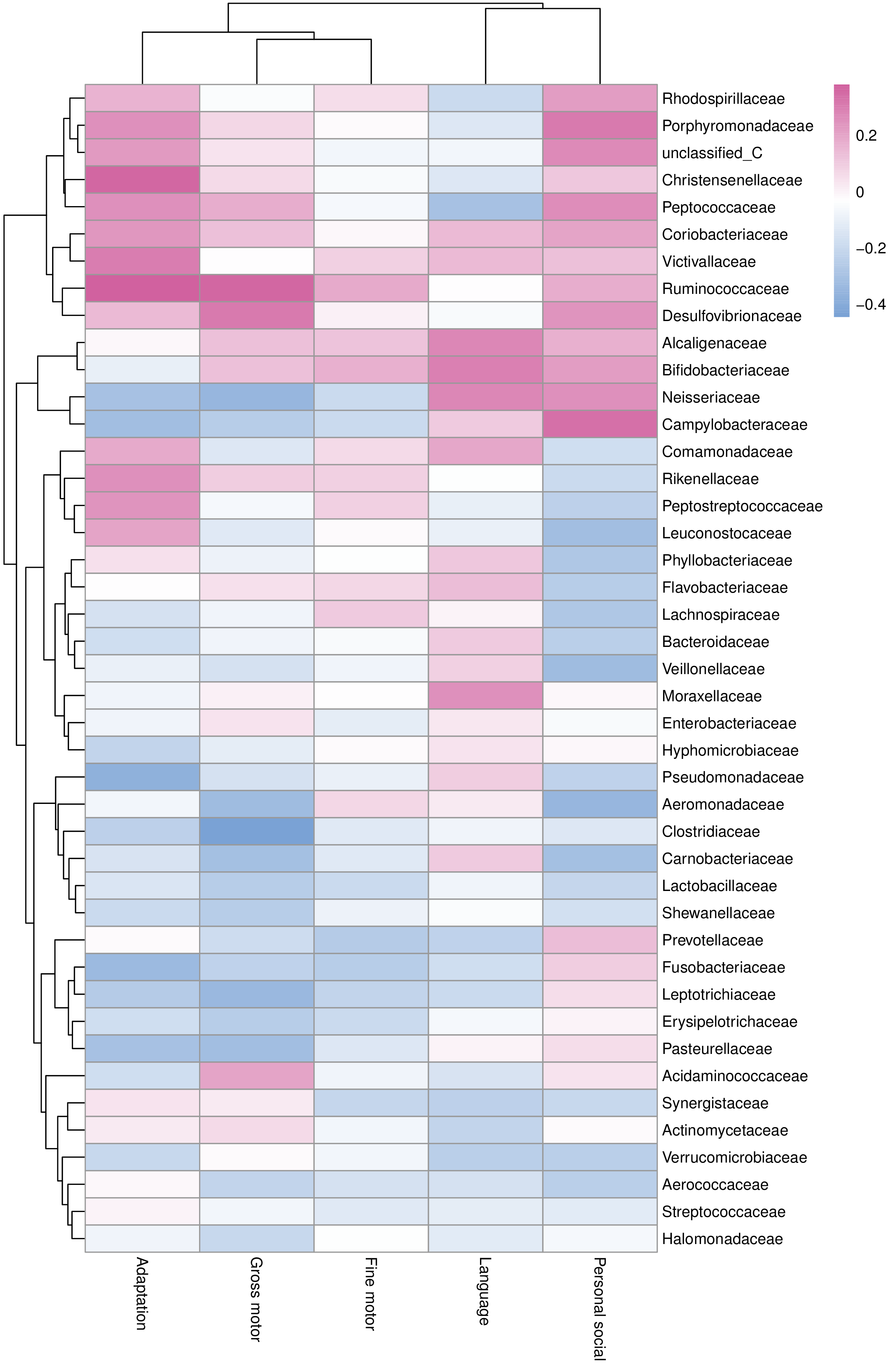


**Fig. S4** Heatmap of the Spearman correlation coefficients between scores of five GDI behavior domain and abundance of 43 GM families. Color-coded with blue for minus lower coefficient and red for positive higher coefficient. Dendrograms present clustering of GM families (rows) and GDI behavior domains (columns) which is based on hierarchical clustering with Euclidean distance metric and average linkage.

**Fig. S5** Heatmap of the Spearman correlation coefficients between scores of five GDI behavior domain and abundance of 102 GM genus. Color-coded with blue for minus lower coefficient and red for positive higher coefficient. Dendrograms present clustering of GM genus (rows) and GDI behavior domains (columns) which is based on hierarchical clustering with Euclidean distance metric and average linkage.
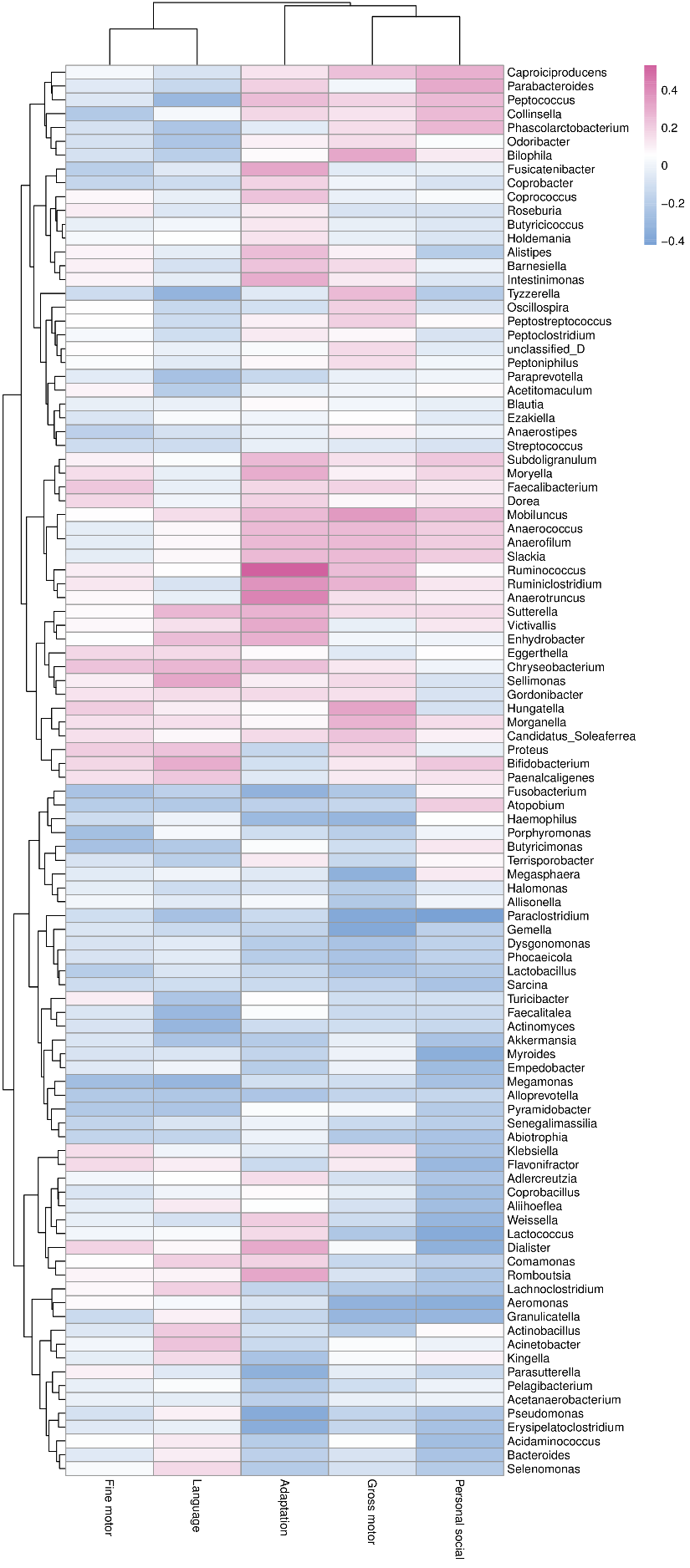


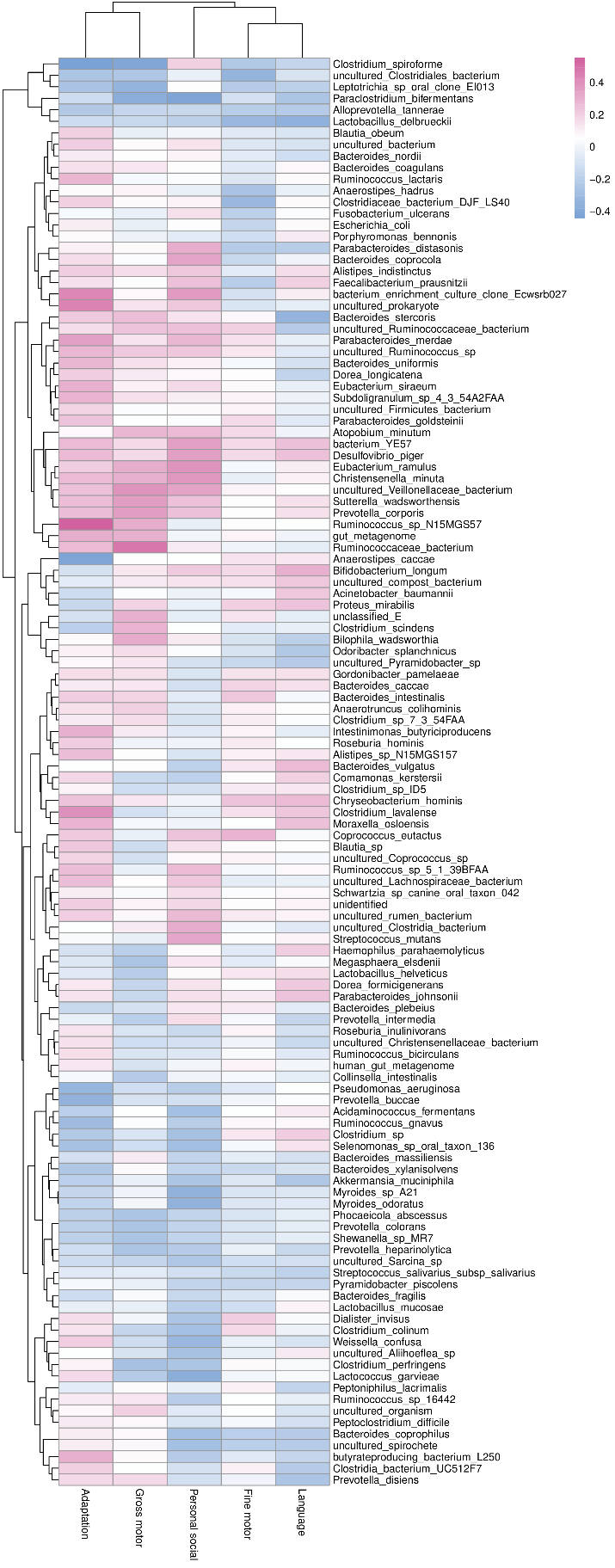


**Fig. S6** Heatmap of the Spearman correlation coefficients between scores of five GDI behavior domain and abundance of 124 GM species. Color-coded with blue for minus lower coefficient and red for positive higher coefficient. Dendrograms present clustering of GM species (rows) and GDI behavior domains (columns) which is based on hierarchical clustering with Euclidean distance metric and average linkage.


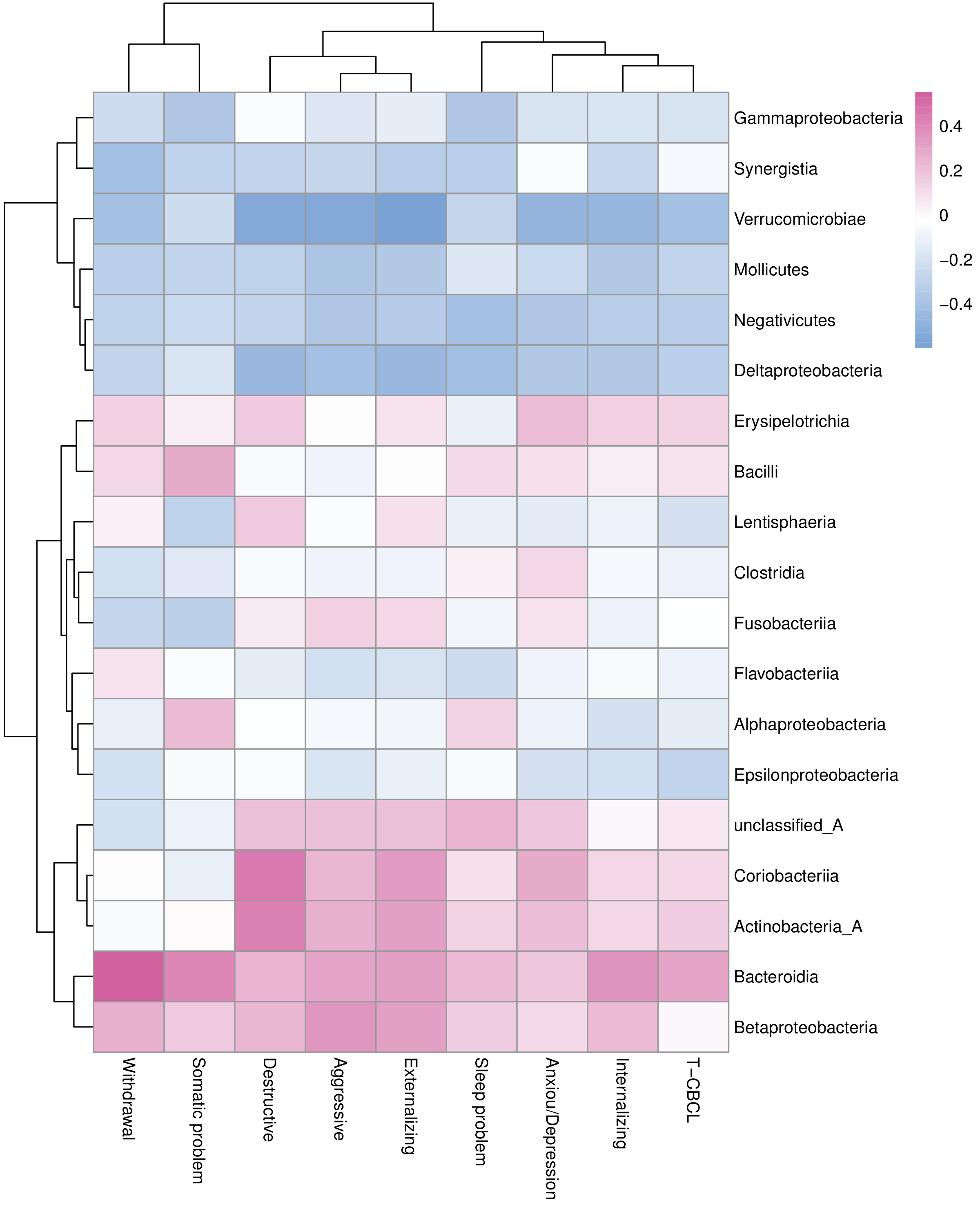


**Fig. S7** Heatmap of the Spearman correlation coefficients between scores of six core syndromes, two broadband syndromes and total Score of CBCL and abundance of 19 GM classes. Color-coded with blue for minus lower coefficient and red for positive higher coefficient. Dendrograms present clustering of GM classes (rows) and CBCL syndromes (columns) which is based on hierarchical clustering with Euclidean distance metric and average linkage.


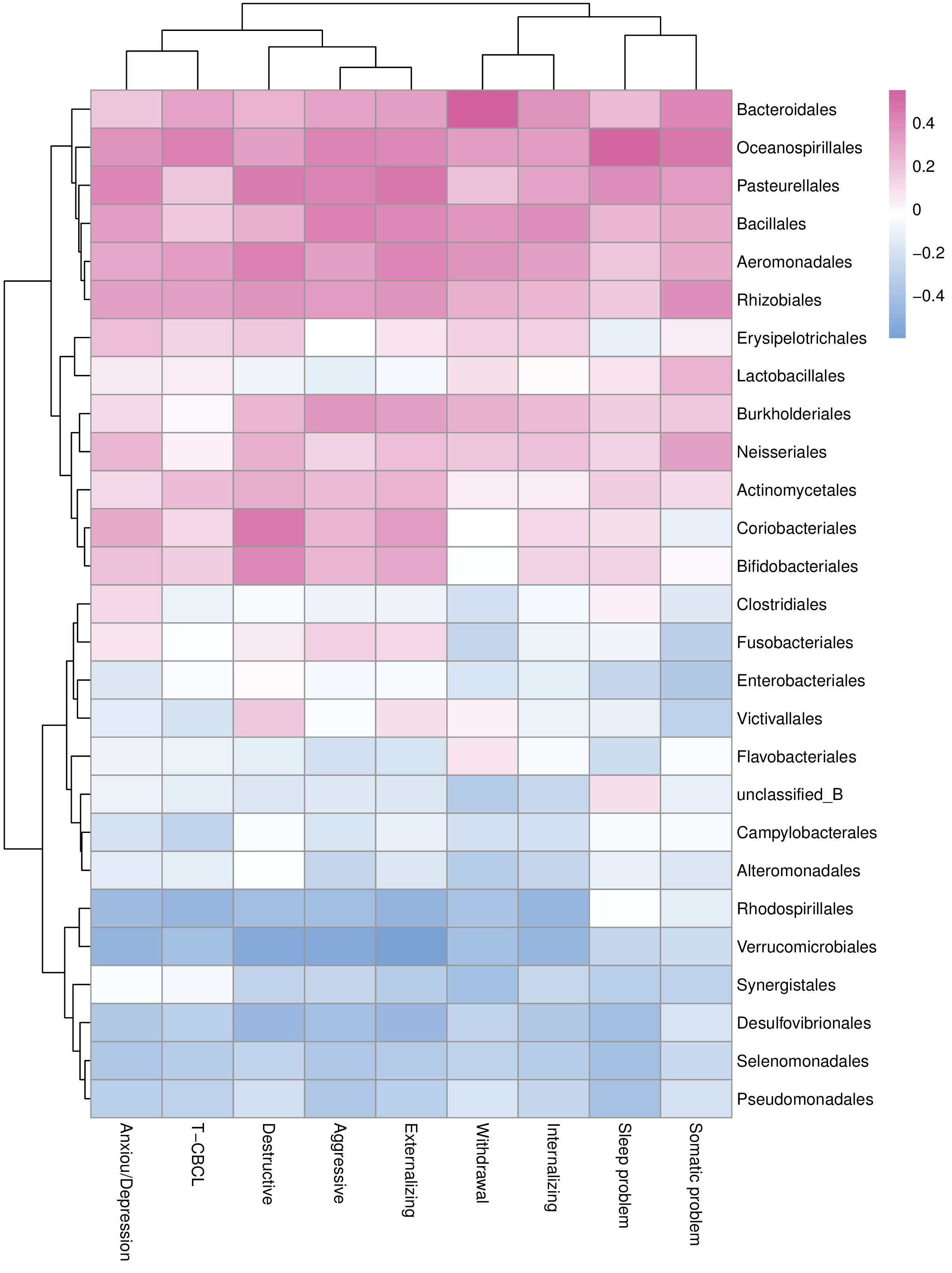


**Fig. S8** Heatmap of the Spearman correlation coefficients between scores of six core syndromes, two broadband syndromes and total Score of CBCL and abundance of 27 GM orders. Color-coded with blue for minus lower coefficient and red for positive higher coefficient. Dendrograms present clustering of GM orders (rows) and CBCL syndromes (columns) which is based on hierarchical clustering with Euclidean distance metric and average linkage.


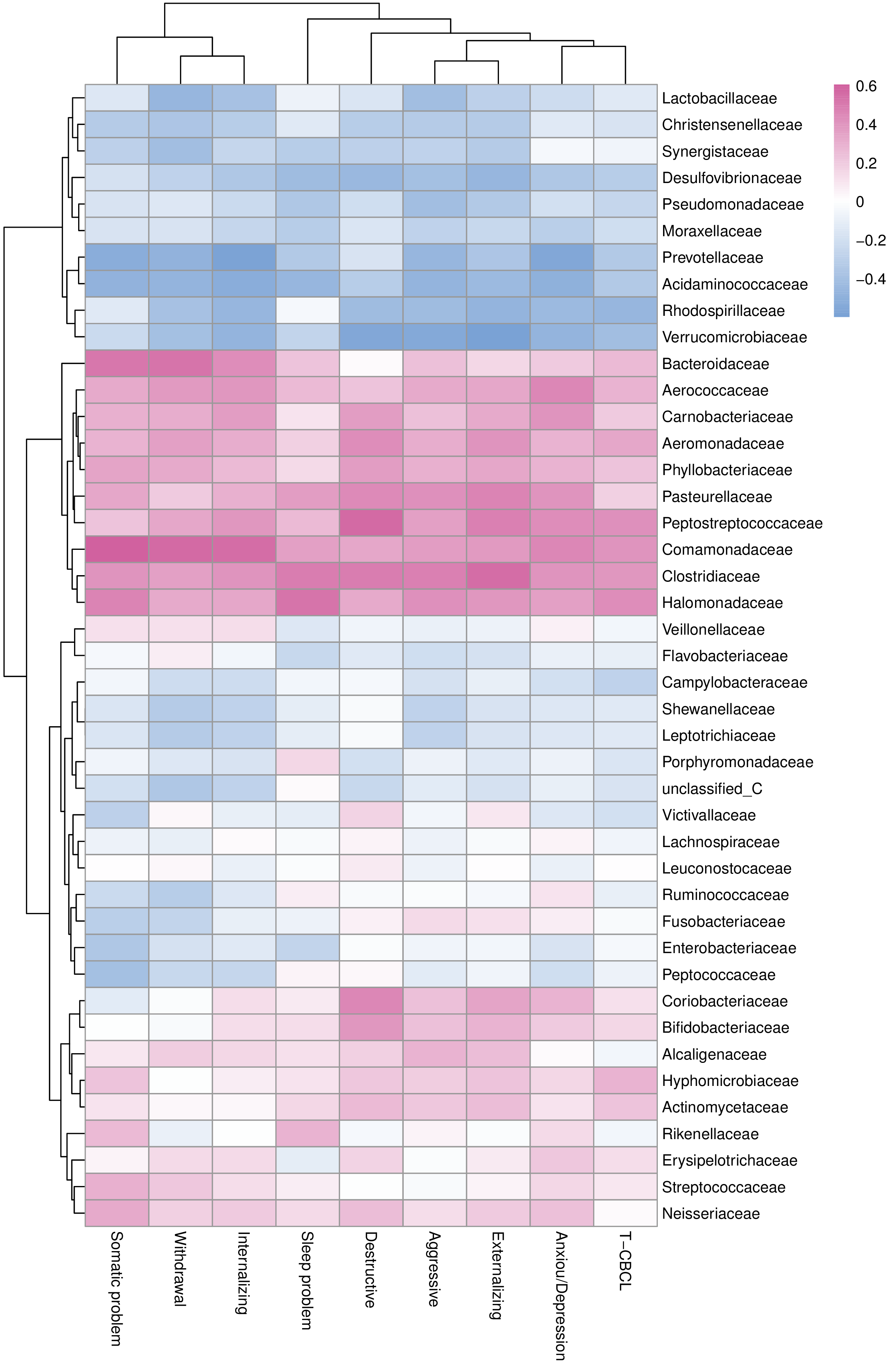


**Fig. S9** Heatmap of the Spearman correlation coefficients between scores of six core syndromes, two broadband syndromes and total Score of CBCL and abundance of 43 GM families. Color-coded with blue for minus lower coefficient and red for positive higher coefficient. Dendrograms present clustering of GM families (rows) and CBCL syndromes (columns) which is based on hierarchical clustering with Euclidean distance metric and average linkage.


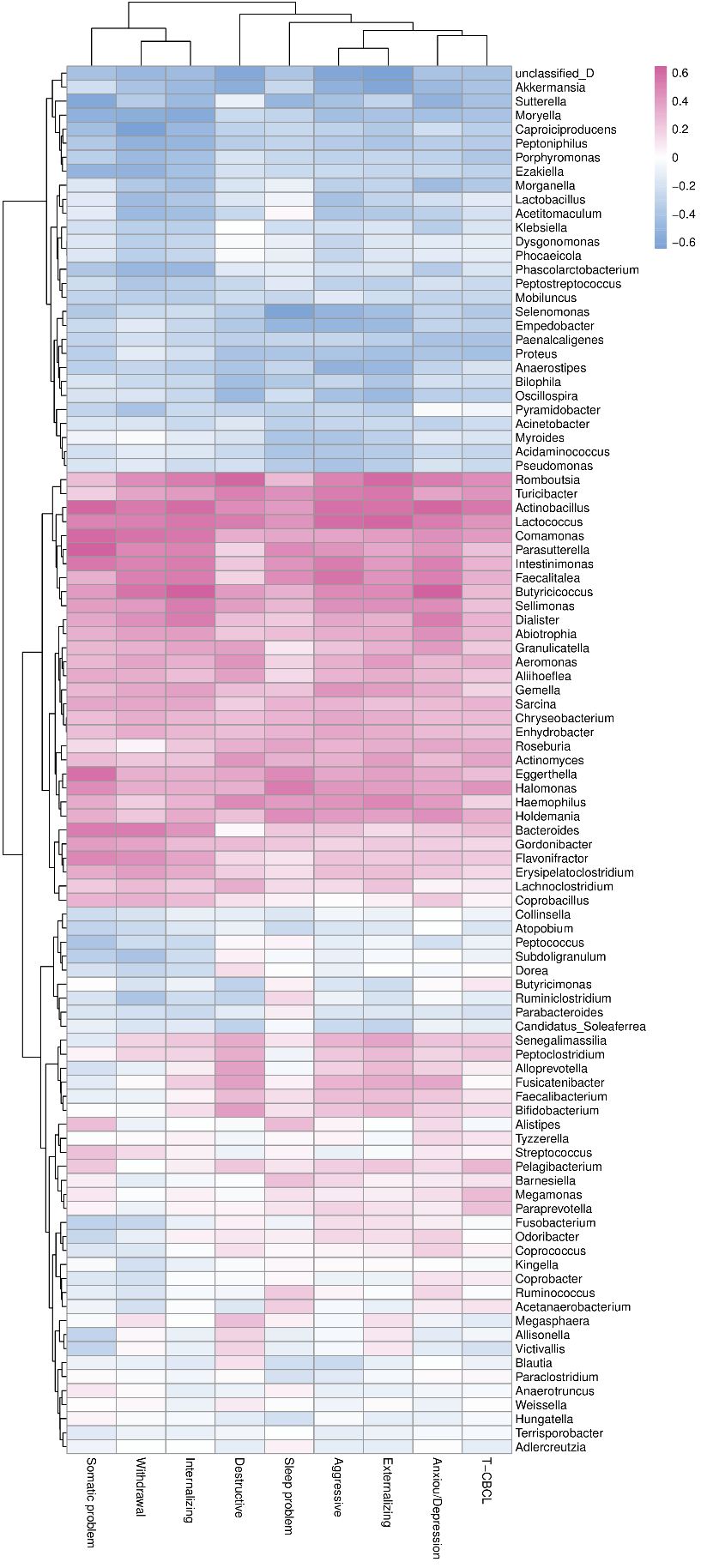


**Fig. S10** Heatmap of the Spearman correlation coefficients between scores of six core syndromes, two broadband syndromes and total Score of CBCL and abundance of 102 GM genus. Color-coded with blue for minus lower coefficient and red for positive higher coefficient. Dendrograms present clustering of GM genus (rows) and CBCL syndromes (columns) which is based on hierarchical clustering with Euclidean distance metric and average linkage.


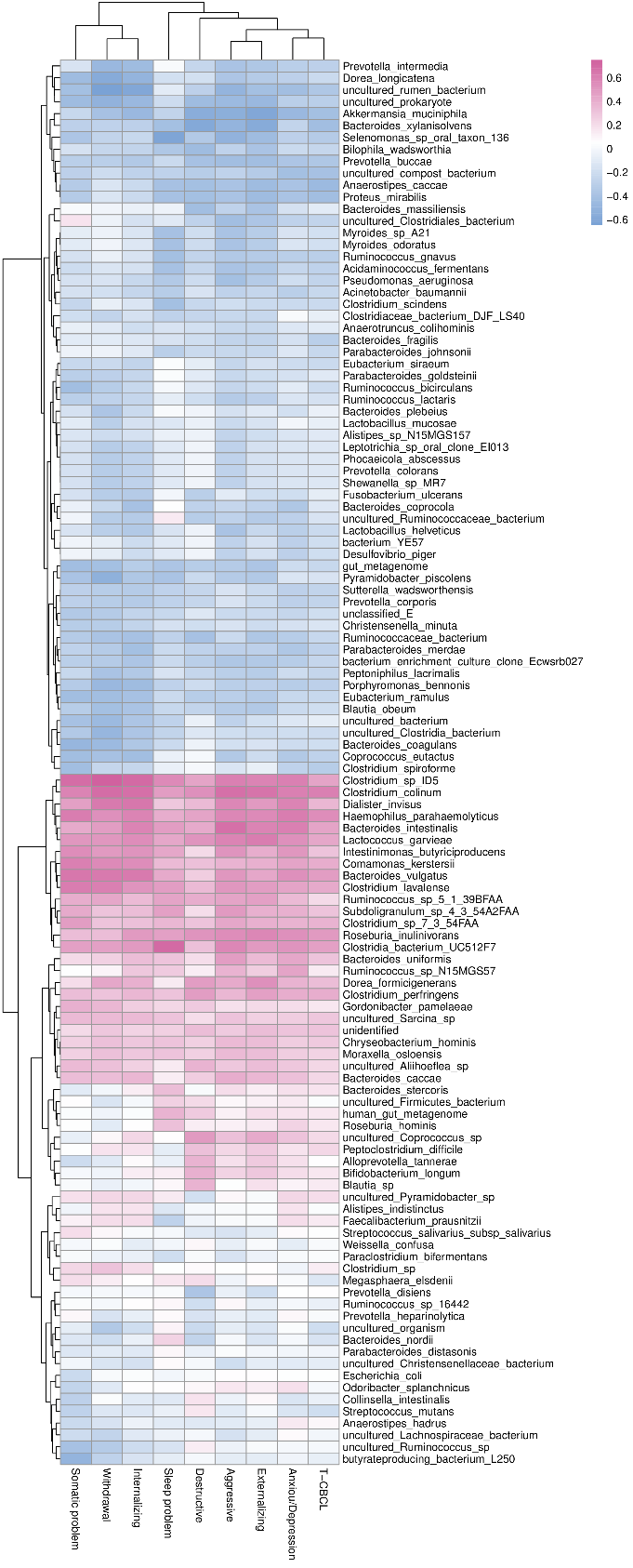


**Fig. S11** Heatmap of the Spearman correlation coefficients between scores of six core syndromes, two broadband syndromes and total Score of CBCL and abundance of 124 GM species. Color-coded with blue for minus lower coefficient and red for positive higher coefficient. Dendrograms present clustering of GM species (rows) and CBCL syndromes (columns) which is based on hierarchical clustering with Euclidean distance metric and average linkage.
